# Surgical Management of Anterior Glottic Webs

**DOI:** 10.3389/fped.2020.555040

**Published:** 2020-10-19

**Authors:** I-Chun Kuo, Michael Rutter

**Affiliations:** ^1^Division of Pediatric Otolaryngology, Aerodigestive and Esophageal Center, Cincinnati Children's Hospital Medical Center, Cincinnati, OH, United States; ^2^Department of Otolaryngology-Head and Neck Surgery, Chang Gung Memorial Hospital, Taoyuan, Taiwan

**Keywords:** congenital glottic web, congenital subglottic stenosis, laryngeal keel, laryngeal atresia, anterior commissure

## Abstract

Congenital webs are rare and represent <5% of all congenital laryngeal anomalies. They are usually a partial laryngeal atresia rather than a true web, and present as a thick and fibrotic web with subglottic extension and associated subglottic stenosis. All patients with a congenital anterior glottic web should be evaluated for chromosome 22q11.2 deletion syndrome. Management strategies are mainly based on the severity of airway obstruction and the anatomical extension of the webs. Simple division of the web endoscopically may be adequate for rare thin webs, however, an open approach is usually warranted for thick glottic webs regardless of Cohen grades. Open repair can be either with keel placement or reconstruction of the anterior commissure.

Laryngeal webs are either acquired or congenital. Congenital webs are uncommon and represent fewer than 5% of congenital laryngeal anomalies. Congenital webs result from a disruption of autolysis of the laryngotracheal groove during 10th week of embryogenesis. Congenital webs vary in size and thickness based on the interruption of the embryologic recanalization process. Therefore, most congenital webs are a form of laryngeal atresia rather than a true web, and present as a thick and fibrotic web with subglottic extension resulting a subglottic stenosis with a small cricoid. In lateral xenography of the airway, the appearance is termed a “subglottic sail” (Figure 1). Anterior glottic webs are the most common type and comprise more than 95% of cases (1).

**Figure 1:**
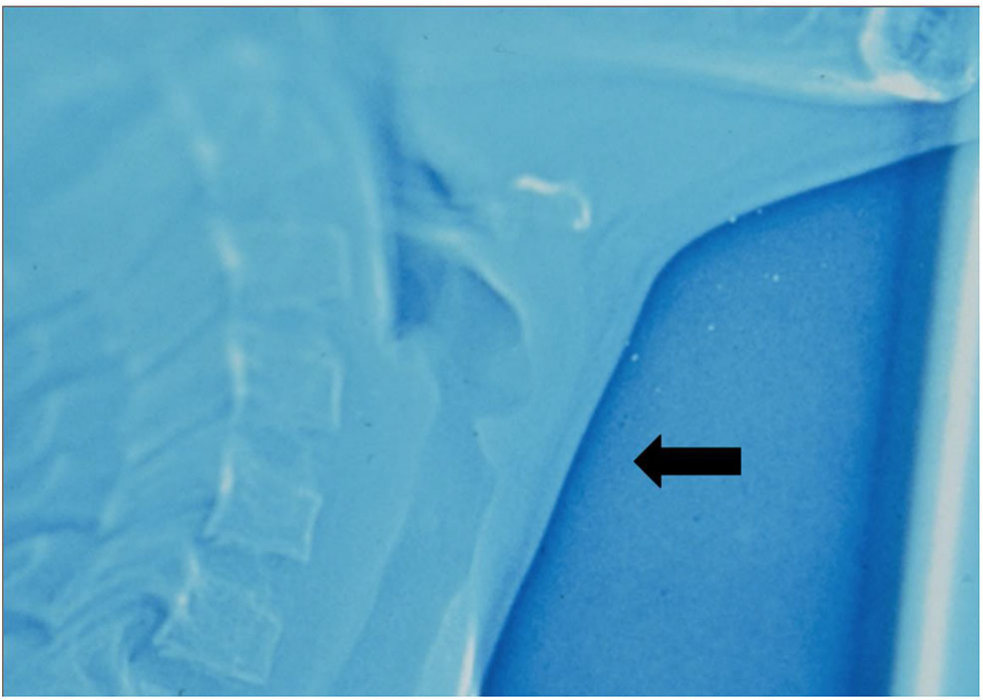
“Subglottic sail” appearance in xenograph.

While there is no single defined gene that results in congenital glottic webbing, there is a significant association between anterior glottic webs and chromosome 22q11.2 deletion syndrome (velocardiofacial syndrome and DiGeorge syndrome). Approximately 65% of patients presenting with an anterior glottic web will have chromosome 22q11.2 deletion syndrome (2–5), and as the web may be the only early manifestation, it is recommended to refer all patients with congenital anterior glottic webs for genetic assessment.

Children born with congenital webs always have an abnormal or even absent cry at birth, and may present with airway obstruction, according to the thickness and location of webs. In an infant presenting with significant airway compromise in the first few days of life, the web is likely severe, and will require emergent airway intervention. Infants are remarkably tolerant of congenital airway compromise, and even patients with severe glottic webbing may initially show only mild airway symptoms, which then exacerbate over the first few months of life. Airway symptoms of severe webbing including biphasic stridor and retractions, which exacerbate when upset or feeding. In more severe cases, failure to thrive, apnea, and cyanosis are characteristic.

Initial evaluation should include awake flexible laryngoscopy to excludeother pathologies, such as laryngomalacia or vocal cord palsy. For definitive evaluation rigid bronchoscopy is recommended, with both the severity of the web and its subglottic extension being assessed. Flexible bronchoscopy offers an excellent view of the anterior commissure, and is therefore complimentary; whereas rigid bronchoscopy better evaluates the degree of subglottic stenosis, and angled (70°) telescopes may provide superior images of the web. In children with a severe web, bronchoscopy should be performed with care, to avoid further compromising an already compromised airway, and spontaneous ventilation with the infant maintaining his or her own airway is preferable to intubation or emergent tracheotomy (6).

Management strategies are mainly based on the severity of airway obstruction and the anatomical extension of the webs (7). The anterior glottic webs are classified by the percentage of vocal cord involvement and the presence of subglottic extension which postulated by Cohen (8) in 1985.

According to the Cohen classification, a type 1 glottic web is a thin web with <35% of glottic involvement. Type 2 involves 35~50% of glottis. A type 3 web, has a 50~75% glottic involvement with anterior cricoid cartilage extension resulting in subglottic stenosis (SGS) formation. And a type 4 web involves 75~90% of the glottis with cricoid extension and associated SGS.

Infants with a type 1 web may present no respiratory symptoms but hoarseness whereas type 2 web causes mild airway symptoms and a weak cry. For a rare true gossamer-thin anterior glottic web, it may never be formally diagnosed, as intubation for airway stabilization may lyse the web and completely resolve the problem. In the rare thin type 1 and 2 anterior webs, simple division of the web endoscopically with a cold instrument (ex. sickle knife) is simple and effective, usually with the baby suspended on a small Lindholm laryngoscope. Excision can also be done with CO2 or KTP laser. Topical application of mitomycin-C is aimed to reduce scar formation, but evidence suggests that mitomycin-C may delay instead of preventing restenosis (9). Whereas, for thick glottic webs regardless of Cohen grades, open approach is usually warranted instead of endoscopic methods (10) as these webs have a strong tendency to recur after endoscopic division.

The timing for repair is mainly based on the severity of airway symptoms. For a severely compromised airway, intervention may either be early repair or tracheotomy placement with late repair. For mild or moderate webs without clinical airway compromise, late repair is preferable. A larger larynx makes surgery technically much easier. Late repair is typically performed by age 4 years, to improve voice quality before school age. Repair may be performed as a single- or double-staged procedure, depending on the experience of intensive care facilities and whether a tracheostomy is already present. A double-stage procedure with a suprastomal stent left in for a longer period is usually warranted for more severe subglottic stenosis, and in 22q11.2 deletion syndrome, especially the DiGeorge variant, post-operative edema may be a feature for months.

External approaches can be either reconstruction of the anterior commissure or open keel placement open. Open keel placement is reasonable for a severely scarred web (congenital or acquired) and requires a complete laryngofissure to adequately expose the larynx by splitting the thyroid cartilage under endoscopic guidance for ensuring midline approach. To facilitate meticulous midline placement of the laryngofissure, we recommend partially incising (grooving) the thyroid cartilage between the superior and inferior thyroid notches. When the web involves the subglottis as a “sail” (Figure 2) extending to the lower border of the cricoid cartilage, the laryngofissure is carried through the cricoid cartilage (Figure 3). To evaluate whether an anterior cartilage graft is required, a segment of age-appropriate endotracheal tube is placed through the split cricoid. If the subglottis cannot close comfortably over the endotracheal tube, a graft is used to repair the associated subglottic stenosis. The graft is inserted at cricoid level with its superior aspect being the upper border of the cricoid cartilage, and an appropriate size laryngeal keel is trimmed and inserted between the upper border of the anterior cricoid graft (if such a graft is present) to the superior thyroid notch. For preventing epiglottic petiole prolapse, the keel should be placed below the petiole insertion.

**Figure 2:**
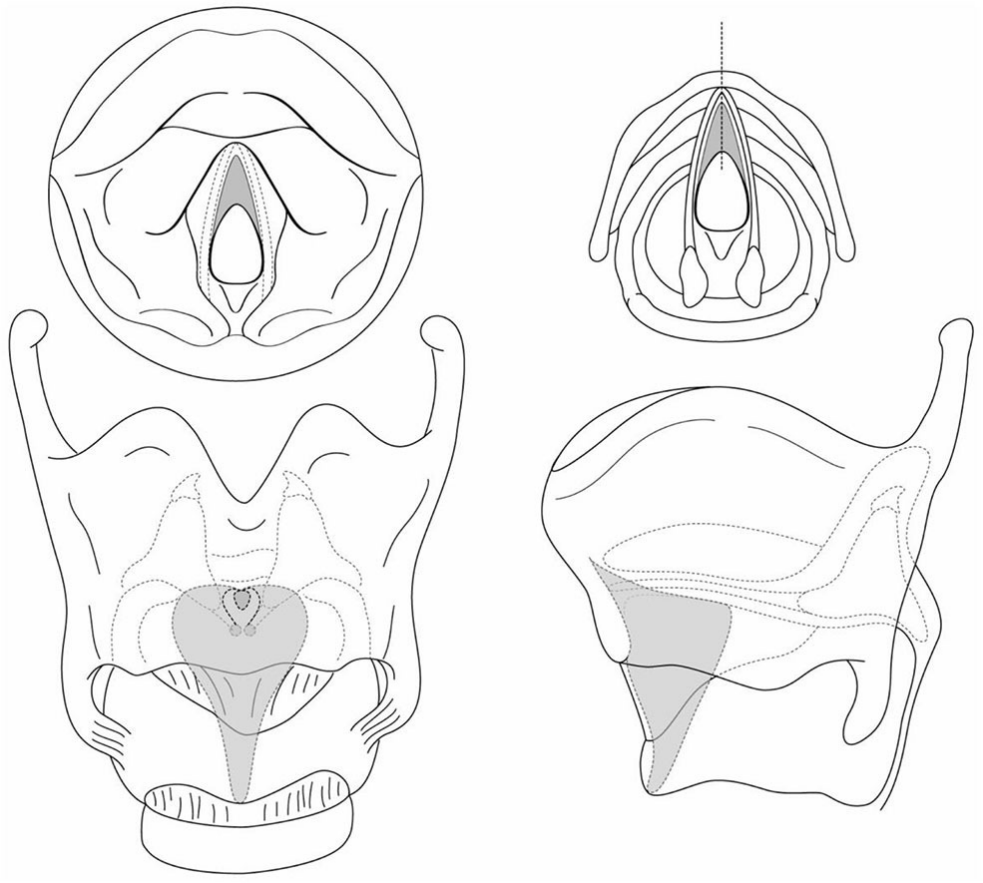
The laryngeal web with subglottic extension appears as a “subglottic sail”.

**Figure 3:**
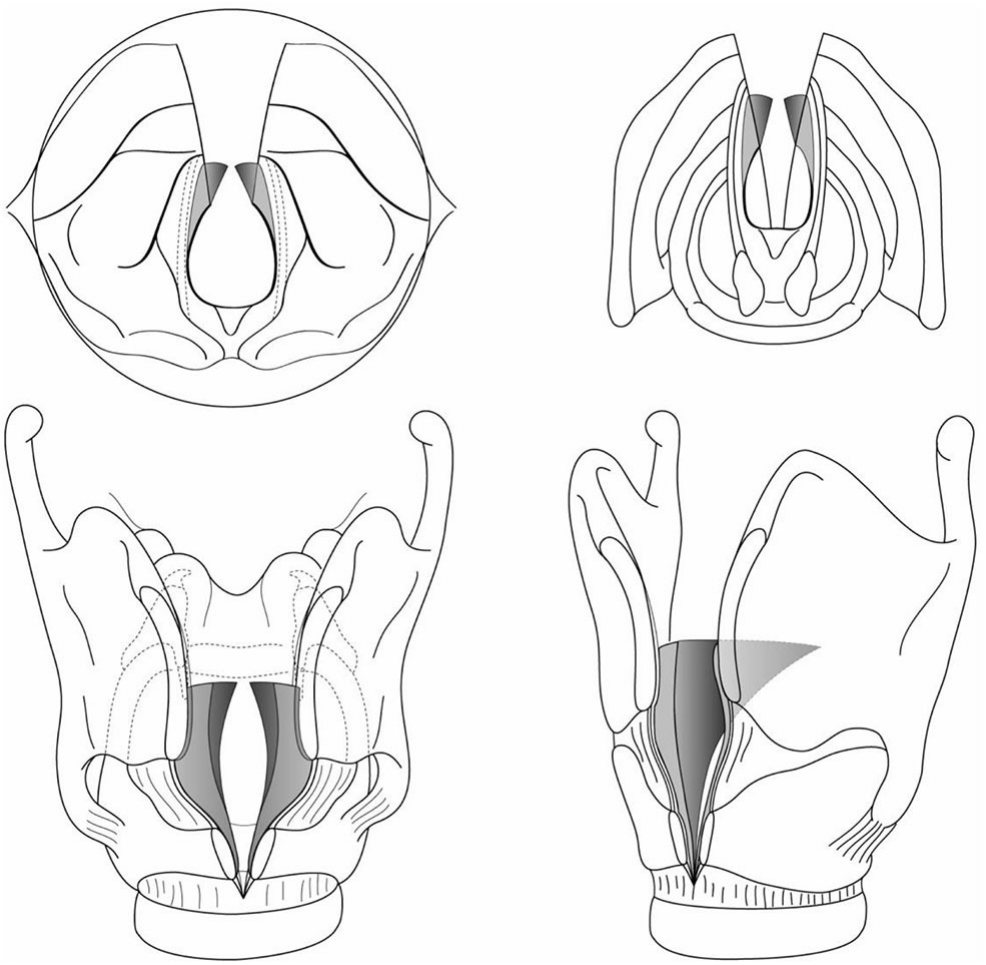
The laryngofissure is down and through the cricoid cartilage.

The infraglottic part of the keel should extend far enough to cover the raw split edges of the web, but should not about the mucosa of the posterior glottis. The keel is sutured into place with a complex suture technique. The airway is then closed with either single- or double-stage procedure. The keel is usually removed within 2 weeks, with longer stenting periods used for more severe webs. Another open procedure with laryngofissure is required for stent removal. Laryngofissure closure requires laterally placed mattress sutures.

There is an endoscopic alternative for laryngeal keel placement which is technically more challenging. It is ideal for minor webs whether congenital or acquired. Tracheotomy or intubation may not be necessarily required. This technique involves initially suspending the larynx with a laryngoscope, and endoscopically dividing the web with a sickle knife (Figure 4). With the patient still suspended, the neck is then prepped, and a small horizontal incision is made over the thyroid cartilage. A 4.0 Prolene suture on a small straight (Keith) needle is then passed through the midline of the lower thyroid cartilage, below the web, and visualized in the airway. The assistant then grasps the needle with a laparoscopic needle holder, and withdraws the needle from the mouth (Figure 5). The surgeon then places a hollow large bore needle through the midline of the upper thyroid cartilage, above the web until it is visualized in the airway. Next, an appropriate laryngeal keel (typically a thin piece of silastic sheet) is used to cover the raw surfaces of the web and passes the straight needle though the inferior and superior borders of the keel in the midline. The small needle is then placed into the lumen of the large bore hollow needle and withdrawn back into the neck incision (Figure 6). The suture is secured over a segment of an intravenous cannula, with multiple suture. The keel is removed 7 to 14 days later, with the neck incision being partially reopened to remove the suture securing the keel (6).

**Figure 4:**
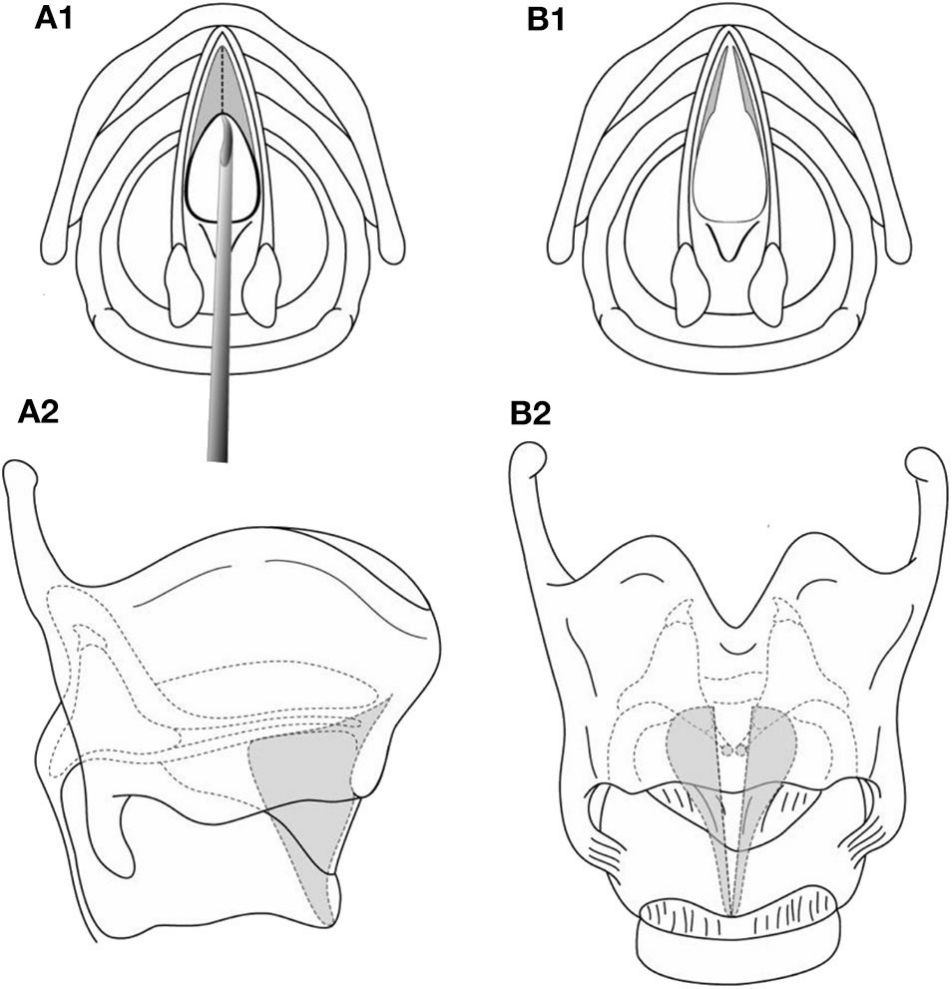
**(A1,A2,B1,B2)** Endoscopic division of an anterior glottic web.

**Figure 5:**
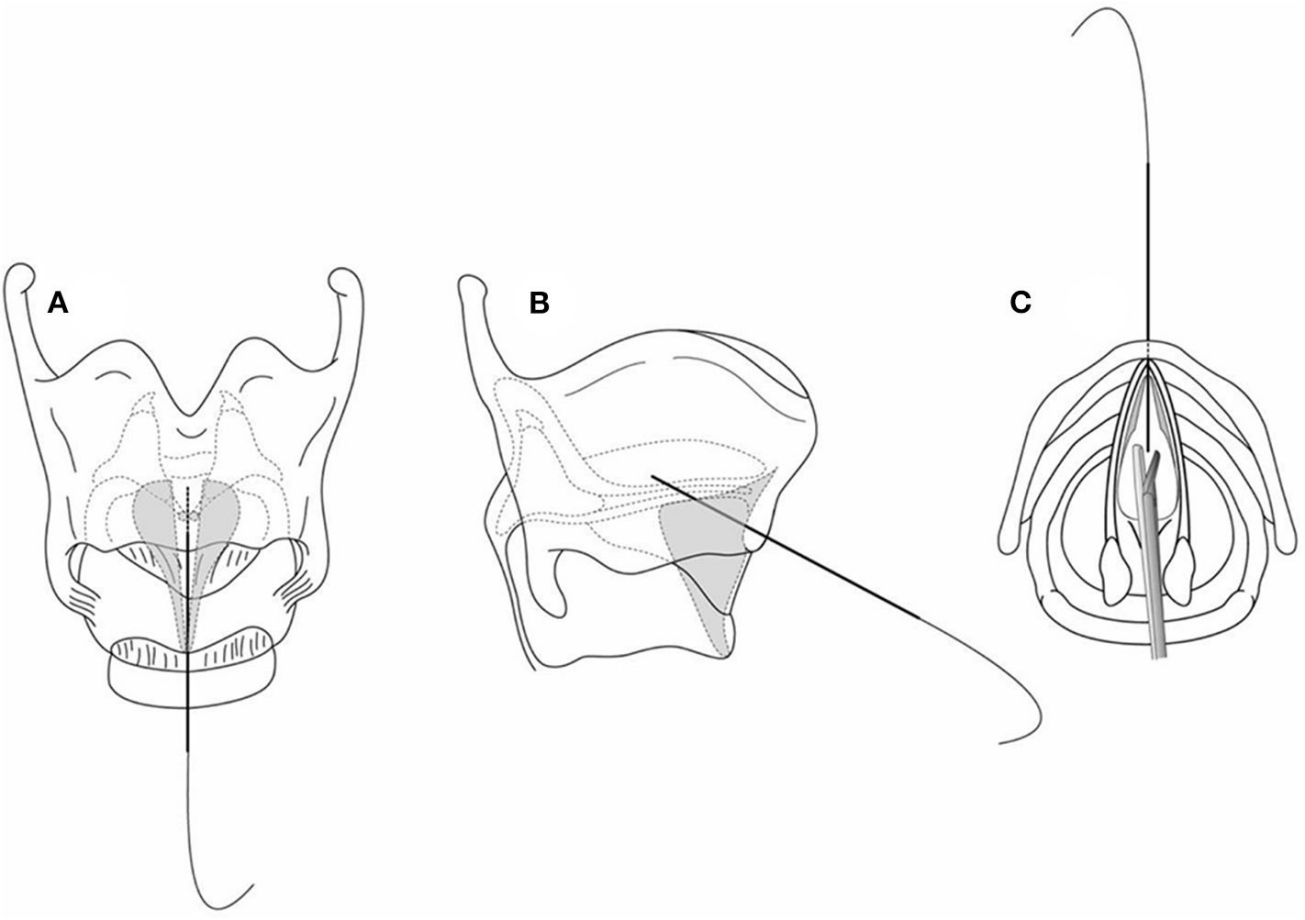
**(A–C)** The suture to secure the web is passed into the larynx from externally.

**Figure 6:**
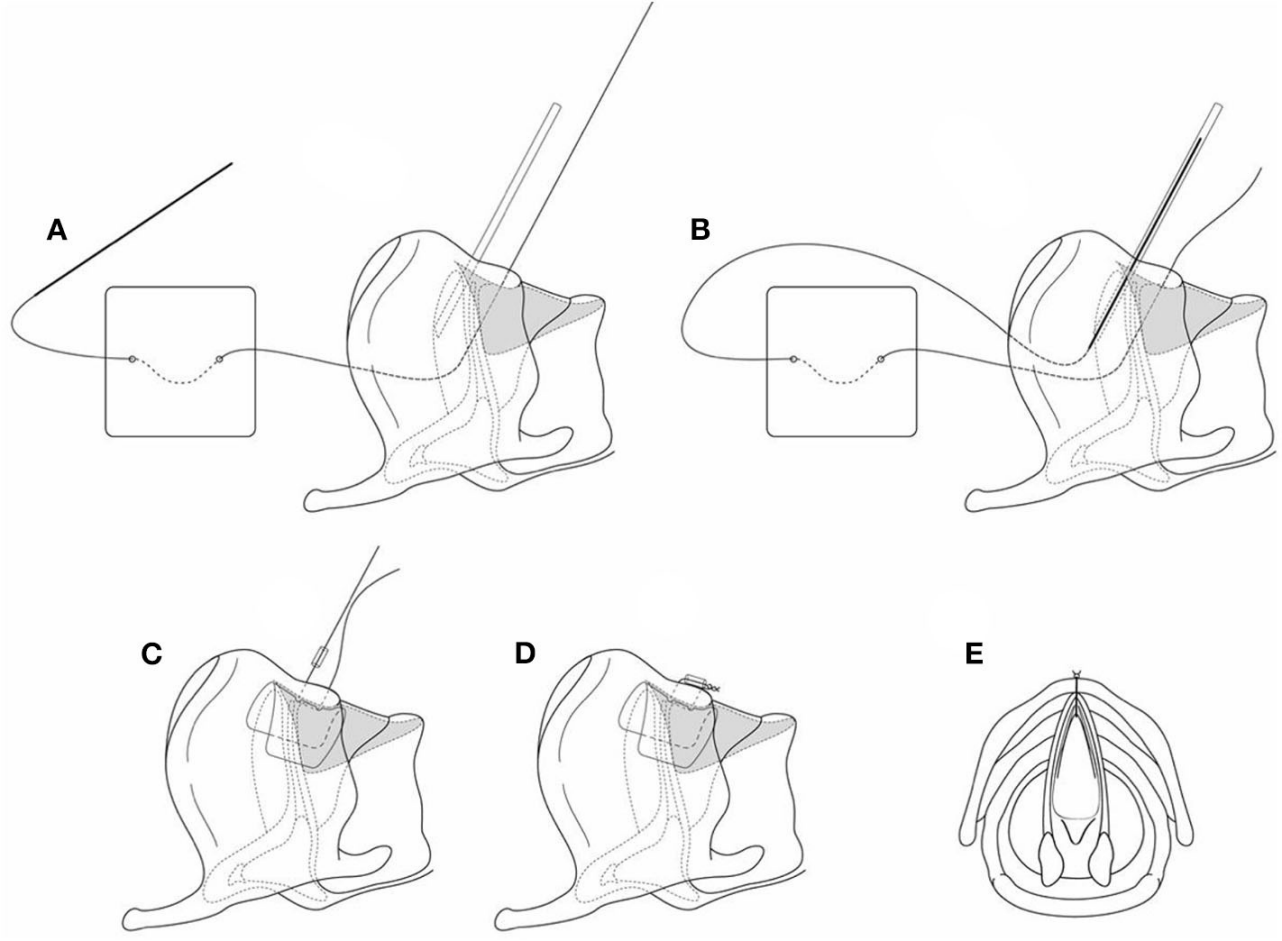
**(A–E)** The silastic keel is inserted on the securing suture, and the suture is passed through a hollow angiocath to exit the larynx, and tied to secure the keel.

An alternative approach is open reconstruction of the anterior commissure, without a keel. Once the laryngofissure is done, if the mucosa at the cut edge overlying the vocal cord is suitably mobile, pexing sutures are used to attach it to the cut edge of the thyroid cartilage, recreating an anterior commissure. 6.0 PDS suture is recommended, on double armed BV-1 needles, with these being placed through the mucosal edge, and then through the thyroid cartilage near the edge of the laryngofissure at the level of the vocal ligament (Figure 7). This ligament lies at the junction of the lower third and upper two thirds of the laryngofissure in an infant. The suture is secured as a mattress fashion. A second pexing suture is placed below the initial suture to further mucosalize the raw incised edge of the web (Figure 8). The same procedure is then performed on the opposite vocal ligament. A segment of age-appropriate endotracheal tube is then placed into airway to determine whether an anterior cartilage graft will be needed for the subglottis. The airway is then closed, with intubation for 2 to 5 days (if this is a single stage procedure). This technique requires mobile vocal mucosa to achieve the mobility to “pex” the vocal cord mucosa back up to the thyroid cartilage, hence it is not suitable as a salvage procedure for a previously injured or operated web with scar formation. The mobile mucosa may provide a better long-term vocal outcome for its potential mucosal wave for vibration.

**Figure 7:**
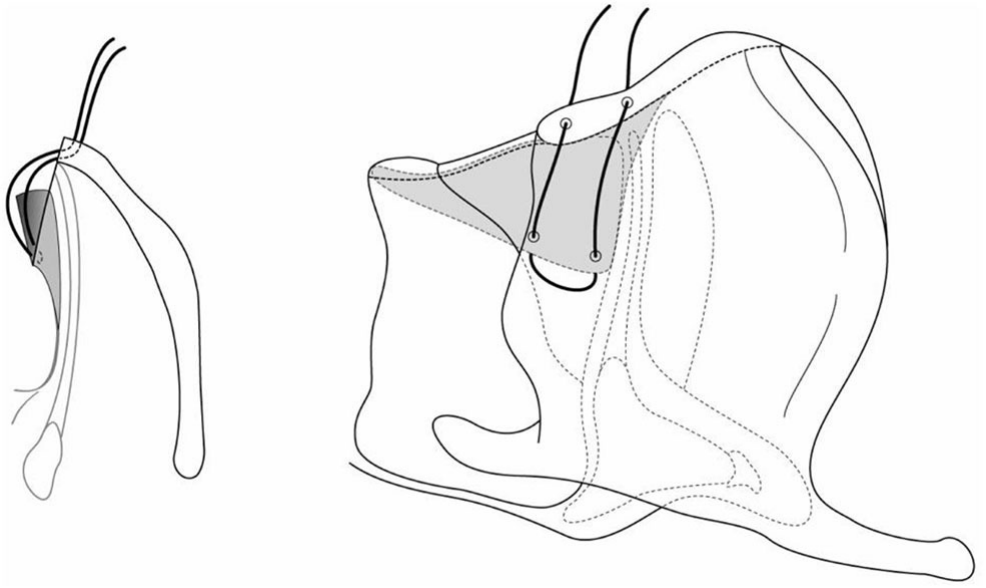
The suture is placed as a mattress fashion.

**Figure 8:**
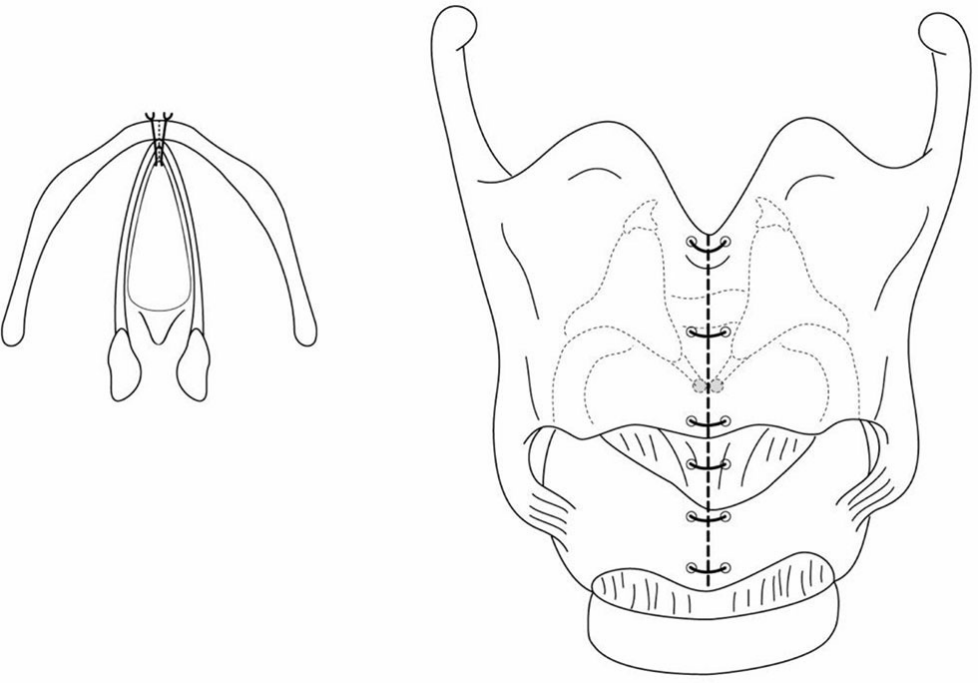
After the pexing sutures are done separately each side, close the larygofissure.

Options for cartilage grafts include thyroid alar and costal cartilage. Thyroid alar is useful for infants, and may be obtained from a superior lateral thyroid alar margin. Costal cartilage is an alternative choice and usually preferable for severe cases.

Total laryngeal atresia generally results in congenital high airway obstructive syndrome (CHAOS) unless there is a concomitant tracheoesophageal fistula. CHAOS is usually a pre-natal diagnosis on ultrasound and magnetic resonance imaging (11) with fetus presenting hydrops, everted diaphragms, mega-trachea, or prune belly syndrome. If recognized prenatally, ex-utero intrapartum treatment (EXIT) procedure to tracheostomy should be planned. As laryngeal atresia patients do not like to have a laryngeal lumen, and as the cricoid tends to be very small, repair is difficult. Repair is recommended after 4 years of age by cricotracheal resection with a complete laryngofissure. A prolonged postoperative stenting for at least more than 6 months is recommended. More than one procedure is typically necessary to achieve decannulation.

The selection of surgical management is usually surgeon dependent. Etiology of laryngeal web doesn't have impact on the choice of surgical procedure and the treatment outcome, whether it is congenital or iatrogenic (12). Surgeons make their decision based on their experience, and the judgement of the severity and complexity for the webs that they are facing. Some surgeons tend to start with endoscopic approach, and preserve open management as salvage procedures for failure cases. Advocators address the issue as open airway reconstruction is usually invasive and may have surgical and/or donor site morbidity. However, Alkan et al. reported higher re-adhesion/recurrence rate is correlated to higher Cohen Classification (12), for type 3/4 laryngeal webs lacking soft tissue for repair, direct open procedures may offer better surgical outcomes. Also, previous laser procedures tend to result in a complicated airway and have negative influence on following open surgical outcomes and more revisions (13).

For the past decades, both endoscopic and open procedures fail to provide promising results. The latest published case series, reported as high as 76 and 89% of recurrence rate for endoscopic and open approach, respectively (14). It once again emphasized the clinical and surgical challenging of laryngeal webs.

In summary, most congenital laryngeal webs are thick, thus open repair is warranted for an optimal outcome. It is recommended that all patients with congenital webs to be tested for 22q11.2 deletion syndrome. Scarred webs, whether congenital or acquired, are better managed with a keel placement, either open or endoscopic. Recreation of an anterior commissure with mobile mucosa of laryngeal webs by open reconstruction may provide a better voice quality even for severe congenital webs.

## Author Contributions

All authors listed have made a substantial, direct and intellectual contribution to the work, and approved it for publication.

## Conflict of Interest

The authors declare that the research was conducted in the absence of any commercial or financial relationships that could be construed as a potential conflict of interest.
